# Severe Postpartum Hemorrhage from Uterine Atony: A Multicentric Study

**DOI:** 10.1155/2013/525914

**Published:** 2013-12-02

**Authors:** Carlos Montufar-Rueda, Laritza Rodriguez, José Douglas Jarquin, Alejandra Barboza, Maura Carolina Bustillo, Flor Marin, Guillermo Ortiz, Francisco Estrada

**Affiliations:** ^1^Critical Care Obstetrics Unit, Complejo Hospitalario, Caja de Seguro Social, Bella Vista, Panama City, Panama; ^2^National Library of Medicine, Lister Hill Center, National Institute of Health, Bethesda, Maryland, USA; ^3^COMIN-FECASOG, 4a Avenida 14-14 Zona 14, Guatemala City, Guatemala; ^4^Hospital Mexico, La Uruca, San Jose, Costa Rica; ^5^Hospital Escuela Suyapa, Boulevard Suyapa, Calle La Salud, Tegucigalpa, Honduras; ^6^Hospital Escuela HEODRA, Avenida Poniente, Leon, Nicaragua; ^7^Hospital Nacional Especializado de Maternidad, 25 Avenida Norte y Final Calle Arce, San Salvador, El Salvador; ^8^Clinica Las Americas, 11 Avenida 11-30 Zona 1, Guatemala City, Guatemala

## Abstract

*Objective*. Postpartum hemorrhage (PPH) is an important cause of maternal mortality (MM) around the world. Seventy percent of the PPH corresponds to uterine atony. The objective of our study was to evaluate multicenter PPH cases during a 10-month period, and evaluate severe postpartum hemorrhage management. *Study Design*. The study population is a cohort of vaginal delivery and cesarean section patients with severe postpartum hemorrhage secondary to uterine atony. The study was designed as a descriptive, prospective, longitudinal, and multicenter study, during 10 months in 13 teaching hospitals. *Results*. Total live births during the study period were 124,019 with 218 patients (0.17%) with severe postpartum hemorrhage (SPHH). Total maternal deaths were 8, for mortality rate of 3.6% and a MM rate of 6.45/100,000 live births (LB). Maternal deaths were associated with inadequate transfusion therapy. *Conclusions*. In all patients with severe hemorrhage and subsequent hypovolemic shock, the most important therapy is intravascular volume resuscitation, to reduce the possibility of target organ damage and death. Similarly, the current proposals of transfusion therapy in severe or massive hemorrhage point to early transfusion of blood products and use of fresh frozen plasma, in addition to packed red blood cells, to prevent maternal deaths.

## 1. Introduction

National and regional statistics of maternal mortality (MM) are crucial to guide program planning of reproductive and sexual health and to develop guidelines for health promotion and international research. These statistics are also essential to guide decision making in entities involved in program development and allocation of financial and human resources. The lack of reliable data on MM has created difficulties in the evaluation of progress towards the Millennium Development Goals no.5 (MDG 5, http://www.undp.org/content/undp/en/home/mdgoverview.html), especially in developing countries where MM rates are known to be high.

Postpartum hemorrhage (PPH) is a major cause of MM around the world with incidence of 2–11% [1–3]. According to the World Health Organization, 10.5% of live births were complicated with PPH, and reports from 2000 show that 13,795,000 women suffered PPH accounting for 13,200 of maternal deaths [4].

The chance of a woman dying during pregnancy and childbirth in Latin America and the Caribbean is 1 : 300 during the reproductive age. In the United States, the probability is 1 : 3,700 [5]. Direct obstetric causes of these conditions are consistent with those recorded in other parts of the world: bleeding (antepartum and postpartum), preeclampsia, sepsis, prolonged labor, obstructed labor, and complications related to abortion [1, 6].

It is estimated that 99% of maternal deaths in the world occur in Africa, Asia, and Latin America, and PPH is the cause of 1/4 to 1/3 of these deaths. The risk of maternal death from PPH is lower in developed countries (1 : 100,000 births in the United Kingdom) compared to developing countries (1 : 16 to 1 : 1,000 births). Seventy percent of the PPH corresponds to uterine atony, and other causes of PPH include retained placental tissue, genital tract trauma, and coagulation disorders that can present as unique or contributing factors [5]. Moreover, the impact of PPH is greater on pregnancy outcomes when analyzing maternal morbidity when considering the 90% of patients who suffered PPH and survived. These patients require highly specialized and costly care during the delivery and postpartum phases [7].

Definitions of obstetrical hemorrhage vary by author, there is not a definite agreement, among authors, and in general, obstetrical hemorrhage is defined as loss of 500 mL of blood after vaginal birth or 1,000 mL after cesarean section [5]. The visual estimate of the amount of bleeding is deemed unreliable and frequently underestimates the magnitude of the problem. Others have used reduced hemoglobin/hematocrit values to evaluate the amount of blood loss, but it is known that there is only a slight correlation of these values during the acute stage of the hemorrhage [8].

Patients with severe hemorrhage are identified by careful expert clinical evaluation and often rely on altered hemodynamic status. Thus, we can consider the diagnosis of serious or severe obstetric hemorrhage to that which exceeds 1,000 mL of blood lost in 24 hours. A review by Carroli et al. [7] reported a prevalence of severe PPH, as loss of >1,000 mL of blood as presented in 1.86% of his patients, his report highlights variations by region but the overall estimate in blood volume agrees with other studies.

Active management of the third stage of labor is the only intervention that has demonstrated significant reduction in MM in patients with PPH. Active management of the third stage of labor includes administration of oxytocin, continuous and gentle traction of the umbilical cord and uterine massage [9]. Despite the reduction of PPH using active management of the third stage, a considerable number of patients develop severe and/or massive bleeding [10].

Treatment of severe hemorrhage secondary to uterine atony should begin with uterotonic agents (oxytocin, methylergonovine, and prostaglandins). Further conservative interventions are intrauterine balloon tamponade (Bakri balloon), uterine compression sutures (B-Lynch), different pelvic devascularization techniques (uterine or hypogastric artery ligation), and vascular occlusion (embolization) [5]. Failure to achieve control of bleeding with the above measures prompts aggressive procedures such as abdominal hysterectomy.

Outcomes in the management of PPH are highly dependent on the availability of trained personnel, surveillance equipment of vital signs, properly equipped operating rooms, blood banks with capacity to provide sufficient supplies for massive blood and blood products transfusions, and specialized anesthesiology services.

The objective of our study was to compile and analyze PPH cases in six countries in Central America during a 10-month period, identify associated factors, and evaluate severe hemorrhage management.

## 2. Methodology

The study was conducted by members of the Research Committee of the Central American Federation of Associations and Societies of Obstetrics and Gynecology (COMIN-FECASOG) in collaboration with an international obstetrician and native Spanish speaking researcher (LR). The study involves thirteen (13) participating institutions from six (6) countries, May 1, 2011–February 29, 2012, as follows:

- Guatemala: Hospital General San Juan de Dios, Hospital Roosevelt,
- Honduras: Hospital Escuela, Instituto Hondureño de Seguridad Social,
- El Salvador: Hospital Nacional de Maternidad, and Hospital de San Miguel, Hospital San Rafael, Hospital Primero de Mayo,
- Nicaragua: Hospital Berta Calderon Roque, Hospital Dr. Oscar Danilo Rosales,
- Costa Rica: Hospital Mexico, Hospital de las Mujeres,
- Panama: Complejo Hospitalario de la Caja de Seguro Social.

The study population is a cohort of vaginal delivery and cesarean section patients with severe postpartum hemorrhage secondary to uterine atony. The study was designed as a descriptive, prospective, longitudinal, multicenter, and comparative study.

The aim of the study was to assess the medical and surgical management of severe postpartum hemorrhage by uterine atony in 13 different hospitals and evaluate maternal outcomes. Assessment is based on the rate of complications and the rate of MM resulting from hemorrhage. The participating maternity services are structured centers of tertiary care (highest complexity level), and all the centers include training programs in obstetrics-gynecology, adult and neonatal intensive care units, blood banks, and specialized anesthesiology services.

The PPH cases were classified according to Benedetti by the degree of hemodynamic compromise [11] as follows:

- grade I (blood loss <15%, without hemodynamic signs or symptoms),
- grade II (blood volume loss of 20–25%, accompanied by tachycardia, tachypnea, and hypotension),
- grade III (blood volume loss of 30–35%, when earlier signs added cold extremities and/or oliguria),
- grade IV (blood volume loss ≥40% and having everything described before plus altered sensory.

We recorded patient data in a log sheet (Table 1). Included in the study were patients with uterine atony in blood loss greater than 20% of blood volume. Information on each patient was recorded and shown on Table 2.

All patients received 5–10 IU of oxytocin immediately after the removal of the product (5–10 IU). Patients with vaginal delivery received oxytocin within the active management of the third stage of labor according to protocol guidelines.

The protocol was reviewed and endorsed by the IRB of each hospital. All data was then entered in an electronic database and processed with Epi Info 7.1.0.6 for statistical analysis (http://wwwn.cdc.gov/epiinfo/7/index.htm).

## 3. Results

Total live births during the study period were 124,019 with 218 patients with severe postpartum hemorrhage (SPHH) (0.17%). Honduras had the highest rate of SPPH due to uterine atony and Guatemala has the lowest rate (Table 2). Total maternal deaths were 8, for mortality rate of 3.6% and a MM rate of 6.45/100,000 live births (LB).

There was no significant difference in the presence of anemia antepartum between patients who died and those who survived (3.4% versus 3.3%, NS).

Table 2 includes the incidence of PPH per 1,000 live births which gives a better view of the problem. El Salvador has more cases but an overall lower incidence when analyzed against live births.

The age range in the study group is as follows: 60.8% of patients were between 20 and 35 years. The average maternal age was 25.31 ± 7.3 years. One hundred eleven patients had vaginal delivery and 107 cesarean section (*N* = 218) (50.9% versus 49.1%, *P* = 0.8). Gestational age at delivery was significantly different between patients who died and those who survived, with a lower mean gestational age among patients who died. Likewise, due to a shorter gestational age, the average fetal weight was lower in patients who died (Table 3).

There was no increased frequency of risk factors for PPH compared with other publications [8]. We have history of post-partum hemorrhage in 5 cases (2.3%), prolonged labor in 18 cases (8.2%), use of utero-inhibitors agents in 13 cases (5.9%), Newborn weight >4.0 kg in 10 cases (4.5%), polyhydramnios in 2 cases (0.9%), and multiple pregnancy in 19 (8.8%) cases.

Forty nine percent of the cases had grade II postpartum hemorrhage, and 16.5% had massive, grade IV postpartum hemorrhage (Table 4).

Twenty-eight patients developed consumptive coagulopathy (Table 5). The 8 maternal deaths were within this group of patients. There were no maternal deaths among the 188 patients without coagulopathy (0/188 versus 8/28; *χ* ^2^, *P* < 0.0001).

All 218 patients required administration of additional uterotonic agents: besides the routine 5–10 IU during the active management of the third stage of labor, additional oxytocin was administered once the severe hemorrhage diagnosis was established. One hundred fifty-three patients required two uterotonic drugs, 48 patients required 3 drugs and in 8 cases the use of four drugs was required (Table 6).

Forty-eight cases failed to respond to medical management and required conservative mechanical procedures to control the hemorrhage. There were seven cases of abdominal packaging, and several patients required more than one maneuver, for a total of conservative maneuvers of 55 (Table 7).

Fifty-two patients persisted with hemorrhage after conservative medical management and/or conservative interventions and required hysterectomy (Table 8). In the hysterectomy group, there were seven maternal deaths, and only one of maternal death patients did not undergo hysterectomy (7/52 = 13.4% versus 1/166 = 0.6%).

Of the 52 hysterectomies, 9 were performed in patients with grade II hemorrhage (0.83%), 19 in patients with grade III hemorrhage (25.6%), and 24 in patients with grade IV hemorrhage (66.6%).

Patients were resuscitated with crystalloids in 162 cases, and in 56 cases, a combination of crystalloids and colloids was used. One hundred seventy patients (77.9%) received transfusion therapy, but only 97 patients complete transfusion therapy (packed red blood cells + platelets + fresh frozen plasma, before an hour of the bleeding been identified.

Among the patients with more severe hemorrhage (*N* = 36), there were 23 cases with adequate management according to the protocol (fluid resuscitation and transfusion therapy) and 13 cases with management that did not adhere to the protocol. The 23 patients who received appropriate management survived, but 8 of the 13 with inadequate transfusional therapy died.

The 8 patients, who died, were classified grade IV hemorrhage and treated according to grade. Similarly, the 8 maternal deaths developed consumption/dilution coagulopathy. Seven of the eight maternal deaths underwent abdominal hysterectomy (Table 9).

## 4. Conclusions

Obstetric hemorrhage remains a major cause of maternal morbidity and mortality. The highest percentages of maternal deaths occur in the immediate postpartum period. Approximately 75% of postpartum hemorrhages are secondary to uterine atony.

The patient population in our study had a lower rate bleeding secondary to uterine atony, with 0.17% of all births, when compared to other publications, as reported by a French group, where patients are evaluated of 106 hospitals, show a higher percentage of severe postpartum hemorrhage (secondary to uterine atony), 0.65% [8].

In UK, a rate of PPH was found to be 6.7/1000 LB [12, 13]. Low et al. in Honduras reported 15% of PPH in births at area rural centers [14]. In Scotland, the rate of severe hemorrhage is estimated at 3.7/1,000 LB [15], higher than that reported in our study population.

Multiple studies have identified several risk factors for uterine atony such as polyhydramnios, fetal macrosomia, twin pregnancies, use of uterine inhibitors, history of uterine atony, multiparity, or prolonged labor. We were unable to find such risk factors in our study population. Anemia during the antepartum period has been a factor associated with increased maternal mortality secondary to hemorrhage, and in our study, we found no such association.

The patients who developed severe hemorrhage due to uterine atony required additional administration of uterotonics, and oxytocin was the most commonly used drug.

In all patients with severe hemorrhage and subsequent hypovolemic shock, the most important therapy is intravascular volume resuscitation, to reduce the possibility of target organ damage and death. Similarly, the current proposals of transfusion therapy in severe or massive hemorrhage point to early transfusion of blood products and the use of fresh frozen plasma, in addition to packed red blood cells, to prevent consumption coagulopathy.

In our study, all patients who were subject to no fluid resuscitation and/or adequate and timely transfusion therapy were considered as “inadequate management.” Patients with inadequate management had a higher rate of maternal mortality.

There were eight maternal deaths, which were recognized as grade IV hemorrhage. All had inadequate management, 6 of which, the management was inadequate in both fluid resuscitation and blood transfusion. Seven of the eight patients did not respond to medical or conservative treatment and required hysterectomy, and all deaths had clotting disorders. The failure of medical management, which forces more aggressive actions, can be a predictor of poor clinical outcome. Patients that required hysterectomy have a higher risk of death; and in our study all but one patient who died was subject to hysterectomy.

The maternal deaths for bleeding (*n* = 8), observed a consistent pattern of cases identified in advanced stages late or severe loss of blood volume, it was necessary in 7/8, hysterectomy, and resuscitation, but transfusion therapy was inadequate. Recent evidence from the literature has demonstrated that during severe hemorrhage, coagulopathy may develop in the early stages of bleeding, even before the consumption of coagulation factors and/or hemodilution [16]. Clinical studies have concluded in turn that transfusion of blood products (packed red cells + platelets + fresh frozen plasma + cryoprecipitate) early (even without the arrival of the lab results) is associated with a better prognosis in patients and less mortality [17, 18]. In our study, this association of adequate fluid resuscitation and early transfusion therapy was evident, especially in patients with massive hemorrhage (grade IV).

Our results support the recommendation to start resuscitation with restoration of intravascular volume with crystalloid or colloid and transfuse the patient with severe hemorrhage with blood products as soon as available, in order to reduce maternal mortality rates.

This study has the same limitations of others with low case count although PPH is one of the primary causes of maternal and perinatal death is fortunately low. Our study has the benefits of a multicenter study, providing the opportunity to improve the statistical power and more strength to the findings and conclusions.

It is almost impossible to compare the conditions and resources of the various participating hospitals. Although all institutions are tertiary level of care with qualified personnel, changes in different countries are difficult to compare and evaluate. Likewise, the availability of blood products can never be guaranteed even in level III centers. There may be more patients with bleeding and maternal deaths outside hospitals included in the study. Hospitals involved are teaching hospital and have residency programs. Most likely, the nonteaching hospital maternal mortality rate is higher.

Some studies have shown an increase in maternal mortality when a severe bleeding event was associated with ante-partum anemia. We do not find this association, and ante-partum anemia was not a factor associated with maternal death when bleeding events occurred in our study patients. Transfusion therapy if it was a difference in maternal death regardless of ante-partum hemoglobin, as was the conclusion of the study.

Hysterectomies in patients with grade II hemorrhages were decisions made at the time by the treating physician and based on the unavailability of another treatment option at that time to stop the bleeding (B-Lynch, Bakri, etc.). Unfortunately, we do not have the exact data on how many units of blood were transfused before making or deciding a hysterectomy.

We could not determine if there was delay in deciding hysterectomy in the patients who died. It is impossible to know if the hemorrhage was in advanced stages (grade IV) when the decision was made and if early invasive intervention could have protected the lives of patients.

Clinical guidelines for management of post-partum hemorrhage are based on expert opinion and low level of evidence. It is important to develop new study protocols in order to determine which interventions would achieve to reduce severe maternal morbidity and mortality. Prospective clinical studies are urgently required to determine whether the transfusion of blood products in the early stages of hemorrhage should be adopted or abandoned. The low incidence of the complication makes it difficult to achieve large study groups, and we advocate more multicenter studies.

## Figures and Tables

**Table 1:** Data recorded for each patient.

History of postpartum hemorrhage	Yes or no
Multiple gestation	Yes or no
Polyhydramnios	Yes or no
Prolonged labor	Yes or no
Active management of the third stage of labor	Yes or no
Using uterotonic agents	Yes or no
Antepartum anemia (hemoglobin <10 g)	Yes or no
Gestational age of the newborn	In weeks
Delivery	Vaginal or cesarean section
Adequate fluid resuscitation	Crystalloids in 3 : 1 proportion to the losses or colloids 1 : 1 Administered the first 30 minutes of identified the bleeding
Adequate transfusion therapy	Six units of blood products (ratio of packed blood cells: fresh frozen plasma: platelets; 1 : 1 : 1) within the first hour after hemorrhage has been identified
Complications secondary to bleeding	Severe anemia, coagulopathy, acute renal failure, ARDS, cardiac arrest
Maternal death	Yes or no

**Table 2:** Distribution of severe postpartum hemorrhage (uterine atony) by country.

Country	PPH cases % of SPPH	Live births	PPH per 1000 live births
Guatemala	29 (13.3%)	34,200	0.85
Honduras	79 (36.2%)	18,892	4.18
El Salvador	55 (25.2%)	36,774	1.49
Nicaragua	36 (16.5%)	20,870	1.72
Costa Rica	8 (3.6%)	8,700	0.92
Panama	11 (5.0%)	4,583	2.40

Total	218 (100%)	124,019	1.75

**Table 3:** Patients' characteristics of severe postpartum hemorrhage.

Maternal age	Total 25.31 ± 7.3 years old Alive 25.27 ± 7.4 years old Dead 26.25 ± 4.2 years old	*P* = 0.7 No statistical significance (NS)
Gestational age at delivery	Total 37.6 ± 3.2 weeks Alive 37.7 ± 2.9 weeks Dead 34.0 ± 7.2 weeks	*P* < 0.01
Newborn weight	Total 2,990 ± 664 g Alive 3,013 ± 646 g Dead 2,308 ± 862 g	*P* < 0.01
Parity	Primipara 123 (56.4%) Multiparous 95 (43.6%)	NS
Simple and multiple pregnancy	Simple 199 (91.3%) Multiple 19 (8.7%)	No MM with multiple pregnancy

*The table compares patients with SPPH, those who survived with those died.

**Table 4:** Case distribution based on severity.

Hemorrhage	Cases	Percentage
Grade II	108	49.5%
Grade III	74	33.9%
Grade IV	36	16.5%

Total	218	100%

**Table 5:** Complications secondary to severe hemorrhage.

Damage to body	Cases	Percentage
Severe anemia	104	47.7%
Coagulopathy	28	12.8%
Acute renal failure	26	11.9%
ARDS	25	11.4%
Cardiac arrest	11	5.0%

Total	194	**∗**

*Sixty (60) patients had more than one organ affected.

**Table 6:** Additional uterotonic used in uterine atony during active management of the third stage of labor.

Uterotonic	Cases
Oxytocin	193
Prostaglandin	131
Ergotamine	118
Carbetocin	40

Total	482

^∗^The 218 patients required at least two or more drugs, that the total number of drugs exceeds the number of patients.

**Table 7:** Conservative interventions.

Interventions	Cases
Ligation of B-Lynch	30
Hypogastric artery ligation	16
Abdominal packaging	7
Bakri balloon	2

Total	55

**Table 8:** Major interventions.

Major interventions	Cases	Percentage
Hysterectomy	52^(1,2)^	23.8
Laparotomy	19	8.7

Total	71	32.5

^(1)^Total hysterectomy: 21. Subtotal hysterectomy: 31.

^(2)^Postpartum hysterectomy: 25, postcesarean hysterectomy: 27.

**Table 9:** Maternal deaths (*n* = 8).

Variables	Number of patients
Antepartum anemia	1
Vaginal delivery	3
Cesarean birth	5
Hysterectomy	7
Grade IV hemorrhage	8
Coagulopathy	8
Liquid handling and transfusions as inappropriate	8
